# Innate endogenous adjuvants prime to desirable immune responses via mucosal routes

**DOI:** 10.1007/s13238-014-0125-1

**Published:** 2014-12-13

**Authors:** Xiaoguang Wang, Delong Meng

**Affiliations:** 1 CAS Key Laboratory of Pathogenic Microbiology and Immunology, Institute of Microbiology, Chinese Academy of Sciences, Beijing, 100101 China; 2 School of Biomolecular and Biomedical Science, University College Dublin, Dublin, Ireland; 3 School of Biology and Environmental Science, University College Dublin, Dublin, Ireland

**Keywords:** mucosal vaccine, adjuvant, innate endogenous molecules

## Abstract

Vaccination is an effective strategy to prevent infectious or immune related diseases, which has made remarkable contribution in human history. Recently increasing attentions have been paid to mucosal vaccination due to its multiple advantages over conventional ways. Subunit or peptide antigens are more reasonable immunogens for mucosal vaccination than live or attenuated pathogens, however adjuvants are required to augment the immune responses. Many mucosal adjuvants have been developed to prime desirable immune responses to different etiologies. Compared with pathogen derived adjuvants, innate endogenous molecules incorporated into mucosal vaccines demonstrate prominent adjuvanticity and safety. Nowadays, cytokines are broadly used as mucosal adjuvants for participation of signal transduction of immune responses, activation of innate immunity and polarization of adaptive immunity. Desired immune responses are promptly and efficaciously primed on basis of specific interactions between cytokines and corresponding receptors. In addition, some other innate molecules are also identified as potent mucosal adjuvants. This review focuses on innate endogenous mucosal adjuvants, hoping to shed light on the development of mucosal vaccines.

## INTRODUCTION

Mucosal vaccination is the direct strategy against infections at the entry portal, where pathogens initiate infections via host mucosae. Compared with other vaccinated methods, mucosal vaccination is desirable for many reasons. Firstly the needle free application provides safety for the vaccinator, vaccinee and community, especially in developing countries (Giudice and Campbell, 2006). Cold chain free delivery reduces the cost on transportation and storage of vaccines. Easy manipulations of mucosal immunizations help to get rid of the dependence on vaccinators, which is flexible for vaccine administration. Moreover, mucosal immunizations increase the compliance with recommended vaccination schedules and reduce the side effects on vaccinee, especially children (Giudice and Campbell, 2006). The most distinct advantage of mucosal vaccination is that the speedy delivery to mucosal sites efficiently elicits mucosal immune responses, especially the secretion of mucosal IgA (Mcghee et al., 1992). Mucosal IgA is prominent to opsonise or neutralize mucosal pathogens and activate complement pathway at the portal of entry (Walker, 2004). In addition, mucosal vaccination also induces systemic immune responses, including serum IgG production and cell mediated responses on distal sites (Kaul and Ogra, 1998).

Mucosal surfaces are rich of immunologic organs, thus all mucosal sites are theoretically able to be used as vaccinated routes. On the basis of logical and practical considerations, oral and nasal routes are focused on the delivery of mucosal vaccines (Levine, 2003). Although oral administration of vaccines has been successfully used against polio, cholera and typhoid fever, intranasal delivery is the most effective way to induce potent and broad mucosal immune responses at multiple mucosal sites (Holmgren and Czerkinsky, 2005). Moreover, oral delivery of large amount of immunogens easily elicits oral tolerance and is challenged by stomach acids upon ingestion, inhibiting the usage of oral vaccines (Holmgren et al., 2003; Levine, 2003). The small surface area of nasal mucosa requires a low amount of antigens and the absence of acidity in nasal environment keeps the stability of antigens (De Magistnis, 2006).

Live or attenuated vaccines are efficient to control infectious diseases. However the safety concerns put live or attenuated vaccines to a debatable dilemma, which hinders the process of clinical applications. It is possible that vaccinated organisms may revert to wild-type or even hypervirulent organisms with its replication in the host. Therefore purified protective antigens were paid more attention to new generation mucosal vaccines. Direct mucosal administrations of soluble antigens only elicit relative low immunogenicity, thus safe and efficacious mucosal adjuvants are often co-administered with antigens to increase immunogenicity of non-living vaccines (De Magistnis, 2006). Furthermore, the adjuvant has been defined as immunopotentiator distinct from the delivery system previously (O’Hagan and Rappuoli, 2004).

Pathogen-derived adjuvants or derivatives have been widely used on the adjuvant system of mucosal vaccines. Although some adjuvants prime to potent and broad immune responses, the toxic threat and side effects of non-human products should be evaluated carefully. For example, a nasal enterotoxin-adjuvanted inactivated influenza vaccine was withdrawn after a short time in the market, due to the usage of adjuvant may cause facial paresis (Mutsch et al., 2004). Apparently the use of innate molecules as adjuvants is more advantageous than other compounds because of less toxicity of innate substances (Holmgren et al., 2003). The properties of the innate endogenous adjuvants in the development of mucosal vaccines for protection against infections will be extensively discussed in the following part.

## CYTOKINES

Cytokines are loosely categorized as signaling proteins, which are released by a broad range of cells. The cytokine network activates and regulates the development of innate and adaptive immunity, balances the humoral and cell mediated immune responses. Thus they are critical in host defense against infections and modulation on immune related diseases (Dinarello, 2000). Immune responses could be initiated by simply adding these signaling molecules either as proteins or encoding DNAs (Holmgren et al., 2003; Cox et al., 2006), highlighting the potential adjuvanticity on vaccinations. A few cytokines in immune systems have been well studied and explored to enhance the vaccine efficacy.

## TYPE I IFNS

Type I interferons (IFNs) have been considered as antiviral agents and primed the development of immune responses (Bracci et al., 2008). Several clinical reports clearly exhibits mucosal delivery of human IFN-α has direct prophylactic and therapeutic effects on cancers, autoimmune diseases and infections caused by influenza virus, RSV, measles virus, papillomavirus, HIV and hepatitis virus (Beilharz et al., 2010). Genes encoding chemokines, cytokines and proteases were significantly up-regulated with mucosal administration of type I IFNs, which are closely associated with antigen processing and lymphocyte activation, migration, apoptosis and protein degradation (Tovey, 2002; Namangala et al., 2006; Tovey et al., 2008). The treatment of IFN-α/β increased the antigen-uptake rate of resident APCs in nasal mucus layer of mice (Bracci et al., 2005). Mucosal administration of IFN-α in low dose efficaciously activated the proliferation of natural killer cells, B cells and T-cell subpopulations in the peripheral circulation (Beilharz et al., 2010). Type I IFNs play the critical signaling role in some well researched adjuvants, including Th1 and CTL polarized types (Proietti et al., 2002; McBride et al., 2006). On the other hand, mucosal application of type I IFNs as immune suppressor efficaciously induces suppressor T cells, and reduces cytotoxic T cells and related cytokine products when encountering autoimmune and inflammatory diseases (Beilharz et al., 2010). Oro-mucosal administration of murine IFN-α significantly reduced the production of allergen specific IgE and the recruitment of eosinophil without detectable toxicity in mice (Meritet et al., 2001).

Intranasal administration of IFN-α/β with an influenza vaccine invoked an efficient humoral response, preventing mice from live influenza virus infection and weight loss (Proietti et al., 2002; Bracci et al., 2005; Bracci et al., 2006). Mice immunized with murine type I IFN and influenza virus A vaccine presented high level of serum and mucosal antibodies, associated with efficacious virus clearance in the lung (Couch et al., 2009). However human trials exhibited that neither serum hemagglutination-inhibiting nor neutralizing antibody responses was significantly elevated in the presence of murine type I IFN. The mortality of mice challenged with lethal dose of influenza virus was significantly reduced by intranasal delivery of murine IFN-β and inactivated influenza vaccine (Cao et al., 1992). Oral administration of murine type I IFN significantly elevated the survival of mice challenged systemically with a lethal dose of encephalomyocarditis virus, vesicular stomatitis virus or varicella zoster virus (Tovey and Maury, 1999). Intranasal immunization with vaccinia virus co-expressing interferon-ε elevated lung VV-specific CD8^+^ CD107a^+^ IFN-γ^+^ population, enhanced lymphocyte recruitment to lung alveoli with reduced inflammation and heightened functional/cytotoxic CD8^+^ CD4^+^ T-cell subset (CD3^hi^CCR7^hi^CD62L^lo^) in murine lung lymph nodes, corresponding to a rapid VV clearance in lungs (Wijesundara et al., 2014).

The immuostimulatory effects of type I IFNs are critically dependent upon the timing and dosage of mucosal administration (Beilharz et al., 2010). For instance, IFNs given prior to immunogen suppressed immunoglobulin production and class switching of B cells. Additionally, the high dose of type I IFNs was immunosuppressive. Meanwhile type I IFNs are regarded as immunosuppressors under the circumstances of autoimmune and inflammatory diseases. Some reports showed the administration of type I IFNs was detrimental to pathogen clearance (Wijesundara et al., 2014). Thus to illustrate the enigmatic roles of type I IFNs as immunomodulators or immunosuppressors requires further work on, but not limited, delivery sites, regulating mechanism, optimal dosages and schedules of type I IFNs, and disease indications and circumstances.

## IFN-γ

IFN-γ, produced primarily by T cells and NK cells in response to antigens or mitogens, plays a critical role in T-cell activation and the establishment of the adaptive immunity (Weaver et al., 2007). Intranasal delivery of murine IFN-γ and inactivated influenza vaccine increased survival of mice challenged with lethal dose of influenza virus (Cao et al., 1992). Further analysis showed the titers of IgA and IgG antibodies from IFN-γ integrated group were enhanced at the early stage of infection, and the titer of HI antibody in the same group was significantly lower than that in mice given vaccine alone at the late stage of infection. Oral administration of murine IFN-γ significantly elevated the survival of mice challenged systemically with a lethal dose of encephalomyocarditis virus (Tovey and Maury, 1999).

Both systemic and mucosal antigen specific IgG1 and IgA antibodies were enhanced by intranasal co-delivery of poly-L-lactide microencapsulated V antigen of *Yersinia pestis* and IFN-γ, however this formulation was detrimental to prevent infections by systemic bacterial challenge in the murine model (Griffin et al., 2002). A low dose of murine IFN-γ was provided in drinking water to adult HAM/ICR mice, following challenged with *Salmonella* *typhimurium* one day later. In comparison with control mice, the application of IFN-γ significantly prolonged survival time and reduced the penetration of Salmonellae into intestinal epithelial cells, development of bacteremia and mortality rate (Degre and Bukholm, 1995). Like type I IFNs, the dosage and immune schedule of IFN-γ should be optimized in mucosal treatment since adverse effects were observed in some reports.

## GM-CSF

Granulocyte macrophage-colony stimulating factor (GM-CSF) enhances the recruitment and activation of APCs (Scheerlinck, 2001). Intranasal administration of human GM-CSF enhanced serum GM-CSF levels and increased total leukocyte counts in rabbits (Watanabe et al., 1995). The levels of pulmonary APCs and cytokines, including IFN-γ and IL-12p40, were significantly elevated in mice administrated with recombinant RSV expressing murine GM-CSF intranasally (Bukreyev et al., 2001). Furthermore GM-CSF-encoding virus shifted virus specific Th2 response to Th1 type. Intranasal co-administration of the HIV DNA vaccine with mouse GM-CSF-expressing plasmid induced high levels of systemic and mucosal antigen specific antibodies, and enhanced delayed type hypersensitivity in mice (Okada et al., 1997). Mice nasally co-immunized with adenovirus vectors encoding murine GM-CSF and amyloid β-protein exhibited predominant antigen specific IgG1 and IgG2b response, suggesting a GM-CSF polarized Th2 immune response (Kim et al., 2005). Recombinant vesicular stomatitis virus expressing murine GM-CSF was highly attenuated in terms of viral dissemination and pathogenesis (Ramsburg et al., 2005). Further analysis showed the addition of genetic GM-CSF enhanced the recruitment of macrophage, CD8 T-cell memory and recall responses in immunized mice.

Mice orally immunized with recombinant rabies viruses expressing GM-CSF exhibited higher number of DCs and B cells in the periphery, higher levels of adaptive immune responses and increased viral resistance than immunization with the parent virus (Zhou et al., 2013). The intranasal administration of GM-CSF expressing attenuated HSV induced protective immune responses against lethal dose challenge of HSV in mice (Parker et al., 2006). Pulmonary DC numbers and secretion of immunoregulatory cytokine IL-12 were significantly elevated by intranasal delivery of murine GM-CSF expressing *Mycobacterium bovis* BCG. (Nambiar et al., 2010). Correspondingly, antigen-specific CD4^+^ T cells increased in both mediastinal lymph nodes and lungs. More importantly, the administration of BCG:GM-CSF significantly reduced the load of infected *M. tuberculosis* compared with mice vaccinated with BCG alone. On the basis of above evidence, GM-CSF facilitates uptake of co-administrated antigens via the recruitment of APCs, thus priming immune responses.

## TNF FAMILY

Tumor necrosis factor (TNF)/TNF receptor (TNFR) superfamily are critically involved in maintaining the homeostasis of the immune system, including beneficial and protective effects in inflammation and host defense (Kayamuro et al., 2009b). Intranasal co-administration of TNF-α along with antigen induced effective antigen-specific systemic IgG and mucosal IgA antibody responses in mice (Kayamuro et al., 2009a). Mucosal application of TNF-α primed to Th2-type immune responses by the analysis of cytokines. The adjuvant activity of TNF-α was proved to be efficacious and safe in mice and the adjuvanticity of TNF-α was associated, at least in part, with increased epithelial permeability. HIV immunogen adjuvanted with TNF-α mutant was used as a mucosal vaccine for induction of antigen specific serum IgG and local or distal mucosal IgA antibody responses when the combination was administrated via the nasal route of mice (Kayamuro et al., 2010a). Other members of TNF family were also tested as mucosal adjuvants in the murine model (Kayamuro et al., 2011). Intranasal delivery of TL1A or APRIL with antigen induced strong antigen specific mucosal and humoral immune responses. Interestingly, the magnitude of immune responses elicited by TNFs was equivalent with that induced by CTB, indicating such candidates are promising to replace toxin-based adjuvants.

## IL-1

IL-1 secreted by various types of cells is a proinflammatory cytokine with a wide range of effects on the host immune system, including up- and down-regulations of many genes related with other cytokines, chemokines, adhesions, cell differentiations and migrations (Thompson and Staats, 2011). Mice nasally immunized with soluble antigens combined with human IL-1α or IL-1β developed humoral and cell mediated immune responses, showing equivalent effect induced by co-administered antigens and CT adjuvant (Staats and Ennis, 1999). Ag-specific serum IgG, vaginal IgG and IgA, systemic delayed-type hypersensitivity, and lymphocyte proliferative responses were significantly elevated with co-administration of antigens and IL-1α or IL-1β. Nasal administration of IL-1α induced potent both systemic and mucosal innate immune responses in mice, dependent on IL-1R1 expression of stromal cells (Thompson et al., 2012). Moreover, the induction of adaptive immunity by IL-1α is dependent on the CD11c^+^ cells. Murine IL-1α exhibited significant adjuvant activity on the induction of HIV-specific serum IgG in mice with nasal route of immunization (Bradney et al., 2002). In addition, nasal immunization of murine IL-1α and HIV immunogen induced significant CTL response and Ag-specific IFN-γ-secreting cells in mice (Staats et al., 2001). Furthermore, intranasal delivery of human IL-1α was proved to be safe and well tolerated in primate models (Egan et al., 2004). Tonsillar application of *Streptococcus sobrinus* protein antigens and human IL-1 elicited higher levels of both mucosal IgA and serum IgG antibodies than protein antigens alone (Kokuryo et al., 2002). Delayed type hypersensitivity to *S. sobrinus*, determined by ear swelling and increasing levels of IFN-γ, was found to be induced only in rabbits immunized with IL-1.

The intranasal administration of murine IL-1α/β with influenza virus hemagglutinin (HA) provoked the increase of Th1- and Th2-type cytokines, systemic IgG and mucosal IgA (Kayamuro et al., 2010b). Moreover mice co-administrated with IL-1α/β and HA were protected from the lethal infection of influenza virus. Nasal administration of human IL-1β adjuvant was as effective as subcutaneous immunization of alum adjuvant on the level of antibodies and protection against lethal dose challenge of *Streptococcus pneumonia* in mice (Gwinn et al., 2010). Like subcutaneous ISA-51 (a water-in-oil emulsion adjuvant) and nasal CTx adjuvants, IL-1β was an efficacious nasal vaccine adjuvant for the induction of protective immunity against a systemic tetanus toxin challenge. Despite of efficacious adjuvanticity of IL-1, the pleiotropic characteristic of IL-1 easily elicits undesired immune responses, requiring well-defined optimized procedures to evaluate IL-1 as mucosal adjuvant.

## IL-2

IL-2, mainly produced by naive T cells encountering antigens, contributes to the growth, proliferation and differentiation of T cells during immune responses. By using live *Lactococcus lactis* as intranasal delivery vector, bacterial expression of murine IL-2 augmented the antigen (tetanus toxin fragment C) specific immune responses (Steidler et al., 1998). Meanwhile recombinant bacteria were not immunogenic in mice. HIV specific delayed type hypersensitivity response and CTL activity were enhanced in mice administrated with genetic murine IL-2 and antigen intranasally (Xin et al., 1998). Moreover DNA vaccination with the IL-2 expression plasmid induced a Th1 dominant immune response. Intranasally delivered plasmid encoding IL-2 shifted TT or CT induced Th2 type immune response to a Th1 type response (McNeela and Mills, 2001). The production of antigen specific IFN-γ, IL-2 and IL-4 was augmented in mice orally administrated with plasmid expressing murine IL-2/Ig (Wierzbicki et al., 2002). Furthermore high level of CTL activity and mucosal/serum antibodies were elicited in the approach of genetic IL-2/Ig. Co-administration of inactivated avian influenza virus with IL-2 strongly enhanced the local immune response after intranasal immunization in a chicken model (Zhang et al., 2009). Recently, recombinant *B. subtilis* spores expressing a fragment of the *Helicobacter acinonychis* UreB protein elicited a strong cellular immune response in orally immunized mice when co-administered with spores expressing IL-2 (Hinc et al., 2014).

Subconjunctival delivery of genetic human IL-2-gD or peptide invoked efficacious neutralizing antibodies and cellular immune responses against HSV (Niethammer et al., 2001; Inoue et al., 2002). Further virus challenge showed the immunization of genetic IL-2-gD and peptide reduced the clinical scores and symptoms of Herpetic Keratitis in mice. In order to improve the efficacy of vaccination in old age population, IL-2 was incorporated into an attenuated, cold-adapted influenza A virus. Intranasal immunization with human IL-2-expressing virus enhanced mucosal and cellular immune responses in both young and aged mice in comparison with administration of the parent strain (Ferko et al., 2006). More importantly, mice immunized with the IL-2-expressing virus were completely protected against a lethal dose challenge of homologous influenza virus. A complicate immunized protocol was applied to the control of Simian/Human Immunodeficiency Virus viremia. The nasal administration of genetic human IL-2 enhanced antigen specific cellular immune response, which provided long lasting protection from disease progression (Bertley et al., 2004).

Inclusion of human IL-2 into the intranasally administered liposomes containing bacterial polysaccharide from *Aerobacter euanicum* and *Pseudomonas aeruginosa* increased titers of bacterial polysaccharide specific sIgA and pulmonary plasma cells (Abraham and Shah, 1992). Following analysis showed intranasal immunization with *P. aeruginosa* polysaccharide and IL-2 significantly reduced the murine mortality from *P. aeruginosa* pneumonia. Mucosal immunization of mice with recombinant *L. lactis* NZ9000 expressing the UreBe-IL-2 conjugated protein elicited more anti-UreB antibodies and more cytokines such as IFN-γ, IL-4 and IL-17, and had a lower *H. pylori* burden and urease activity than control mice (Zhang et al., 2014). To sum up, both Th1 and Th2 immune responses are polarized by the mucosal application of IL-2, thus efficaciously preventing intracellular and extracellular infections.

## IL-4 AND IL-5

Cytokine IL-4 induces the T cell differentiation from naive Th0 cells to Th2 cells, which subsequently produce additional IL-4 in a positive feedback loop. Furthermore, IL-4 participates into the proliferation of activated B-cell and T-cell, and the differentiation of B cells into plasma cells. High levels of systemic and mucosal antigen-specific antibodies were induced in mice immunized genetic HIV antigen and mouse IL-4 intranasally (Okada et al., 1997). Moreover, the IL-4 expression plasmid mainly induced IgG1 subtype in serum, however significantly suppressed the delayed type hypersensitivity and CTL response. Intranasal immunization of poly-L-lactide microencapsulated V antigen of *Y. pestis* and IL-4 induced both systemic and mucosal antigen specific IgG and IgA, however this combination vaccine reduced the survival of mice challenged with systemic bacterial injection (Griffin et al., 2002).

IL-5 produced by Th2 and mast cells stimulates B cell growth, increases immunoglobulin secretion and mediates eosinophil activation. Mice intranasally inoculated with adenovirus expressing murine IL-5 exhibited increased viral specific IgA titers in lung lavage fluid (Braciak et al., 2000). Correspondingly viral specific antibody-secreting cells presented high frequency in lung lymphocytes derived from mice immunized with IL-5 expressing adenovirus. The expression of IL-5 in a recombinant vaccinia virus vector significantly increased co-expressed heterologous antigen specific IgA responses in the lungs of mice given intranasal inocula of the virus (Ramsay and Kohonencorish, 1993). Furthermore, the level of local IgA response to IL-5 administration peaked four-fold higher than that elicited by control virus at 14 days after infection, and was sustained for at least 4 weeks. As Th2 primed cytokines, mucosal administrations of IL-4 or IL-5 facilitate strong humoral immune responses.

## IL-6

IL-6 is a pleiotropic cytokine that regulates both T and B cells (Vansnick, 1990). Tetanus toxin fragment C specific immune responses were primed and augmented by intranasal delivered *Lactococcus lactis* expressing murine IL-6, including serum IgG and mucosal IgA (Steidler et al., 1998). Intranasal inoculation of mice with adenovirus expressing murine IL-6 increased viral specific IgA/G titers in lung lavage fluid (Braciak et al., 2000). Viral specific antibody-secreting cells presented high frequency in lung lymphocytes derived from mice immunized with IL-6 expressing adenovirus. Histological analysis showed a large number of mononuclear cells had accumulated under the inoculation of pIL-6.

IgG1 and IgA antibodies were provoked by intranasal delivery of recombinant V antigen of *Y. pestis* coencapsulated with IL-6, contributing to full protection against systemic bacterial challenge to mice (Griffin et al., 2002). Nasal application of human IL-6 and tetanus toxin (TT) enhanced serum TT specific antibodies and Th2 response, protecting mice from lethal challenge with tetanus toxin (Boyaka et al., 1999). Oral administration of human IL-6 before oral infection with *Campylobacter jejuni* primed *Campylobacter* specific mucosal S-IgA Ab responses (Baqar et al., 1993). IL-6-treated animals shed 3-log-unit reduction in the number of *C. jejuni* within 48 h of infection. Although cell mediated immune responses are rarely reported in mucosal application of IL-6, humoral immune responses are strongly activated in the presence of IL-6.

## IL-10

One role of cytokine IL-10 is to down-regulate inflammations. Intranasal administration of genetic IL-10 diminished Ag-induced delayed type hypersensitivity reactions in associated with the reduction of Ag-specific proliferation and production of Th1 cytokines in mice (Chun et al., 1999). Daily mucosal administration of IL-10 secreting *L. lactis* reduces half symptoms of inductive or spontaneous murine colitis (Steidler et al., 2000). Comparing with intravenous injection, mucosal delivery required much lower dose of IL-10. Further study proved the efficacy and safety of mucosal delivery of human IL-10 secreting *L. lactis* on human trials (Braat et al., 2006). The incipient experimental allergic encephalomyelitis was suppressed in rats nasally administrated with autoantigen and human IL-10 (Xu et al., 2000). Following analysis showed that the approach of IL-10 decreased the proliferation of antigen-specific lymphocyte and antigen-reactive IFN-γ secreting Th1-like cells. As an anti-inflammatory cytokine, adjuvant IL-10 is promising to control autoimmune or inflammatory diseases.

## IL-12

IL-12, naturally produced by DC, macrophages and human B-lymphoblastoid cells, typically induces Th1 response and stimulates NK, T and B cells to produce IFN-γ and TNF-α (Trinchieri, 1995; Watford et al., 2003). Consistently, mRNA transcripts of IFN-γ and IL-10 were elevated in mice immunized with murine IL-12 via nasal route (Arulanandam and Metzger, 1999). Intranasal co-administration of HIV genetic antigen and mouse IL-12-expressing plasmids induced both Th1- and Th2-type responses (Okada et al., 1997). Furthermore high levels of HIV-specific CTLs and delayed type hypersensitivity were enhanced in mice immunized with IL-12 and HIV antigen. Ovalbumin specific antibody levels of serum IgG2a, IgG2b and IgG3 were significantly increased by IL-12 given intranasally, while serum IgG1 was suppressed. Incorporation of murine IL-12 to mice nasally immunized with the combined vaccine of TT and CT increased the secretion of TT-specific CD4^+^ T cells IFN-γ and reduced levels of Th2-type cytokines (i.e., IL-4, IL-5, IL-6 and IL-10), implying the shift of CT-induced immune response toward Th1 type (Marinaro et al., 1999). However, IL-12 enhanced the Th2 type responses in mice given the combined vaccine orally. Murine IL-12 enhanced the production of gD (bovine herpesvirus type 1) specific IgA secreting cells, mucosal IgA/G and serum IgG in intranasally vaccinated mice (Baca-Estrada et al., 1999).

Humoral and cellular responses were enhanced in mice intranasally administrated with plasmid DNA encoding murine IL-12 along with glycoprotein B (gB) DNA (Lee et al., 2003). Recipients of the co-immunization procedure showed greater resistance to vaginal challenge with a lethal dose of HSV-1. In another study, nasal delivery of murine IL-12 and influenza immunogens enhanced productions of both systemic and mucosal antibodies (Arulanandam et al., 1999). Mice immunized with antigen and IL-12 displayed decreased weight loss and significant enhanced survival after lethal dose challenge of influenza virus. The intranasal administration of murine IL-12 expressing attenuated HSV induced protective immune responses against lethal dose challenge of HSV to mice (Parker et al., 2006).

Mice intranasally administrated with murine IL-12 secreting and E7 antigen expressing *L. lactis* were protected from the development of cervical cancer (Bermudez-Humaran et al., 2005). Humoral and mixed Th1/2 immune responses were enhanced in mice immunized with human IL-12 and tetanus toxin intranasally, providing full protection against lethal challenge with tetanus toxin (Boyaka et al., 1999). IL-12 as intranasal adjuvant efficiently prevented pneumococcal infection in murine models (Arulanandam et al., 2001; Lynch et al., 2003; Sun et al., 2007). Mucosal and systemic IgG2a and IgA were enhanced with the inclusion of IL-12, corresponding to the reduction of bacterial load. Intranasal administration of inactivated *Francisella tularensis* and murine IL-12 significantly increased the survival of mice under lethal intranasal challenge dose of *F. tularensis* (Baron et al., 2007). Neonatal mice vaccinated with pneumococcal polysaccharide conjugate vaccine and IL-12 exhibited enhanced IFN-γ dependent mucosal and systemic immune responses to pneumococci, and vaccinated mice were efficiently prevented from both otitis media and invasive infection (Sabirov and Metzger, 2006). Moreover, the dose and toxicity of IL-12 was reduced by the intranasal route comparing with the parental administration route (Huber et al., 2003; Wright et al., 2011). Although IL-12 is regarded as Th1 type cytokine, mucosal application of IL-12 also induces strong Th2 immune responses.

## IL-15

IL-15, mainly secreted by mononuclear phagocytes, induces cell proliferation of both innate and adaptive immune system such as natural killer cells and T cells (Shanmugham et al., 2006). Intranasal expression of murine IL-15 delivered by genetic sequence with HIV antigen increased the HIV-1-specific delayed type hypersensitivity response and CTL activity, and decreased the serum IgG1/IgG2a ratio (Xin et al., 1999a). Moreover, murine lymphoid cells from mice administrated with genetic IL-15 and antigen yielded increasing production of IFN-γ and reducing secretion of IL-4. Intranasal administration of plasmid expressing murine IL-15 and FMDV antigen enhanced mucosal and systemic immune responses in animals (Wang et al., 2008). Mice immunized with IL-15 displayed high level of antigen-specific T-cell proliferation, CTL response and increased production of IFN-γ in both CD4^+^ and CD8^+^ T cells isolated from the spleen and mucosal sites.

Mucosal administration of human IL-15 cDNA enhanced both humoral and cellular immune responses during memory phase of vaccinated mice (Toka et al., 2004; Toka and Rouse, 2005). Intravaginal challenge of HSV-1 showed the application of IL-15 enhanced the Th1-dependent HSV-1-specific response, corresponding to a limited lesion severity and rapid elimination of the infecting virus from the distal mucosal site. Although mucosal administration of IL-15 and SIV vaccine preserved high levels of memory CD4^+^ T cell numbers in rhesus macaques challenged with SIVmac251, the viral load was not reduced in the immunized group (Sui et al., 2011).

## IL-18

IL-18 induces the activation of Th1, NK and CTL cells and production of IFN-γ, and regulates the synthesis of inflammatory cytokines, including IL-2 and IL-12 (Toka et al., 2004). Intranasal immunization of murine IL-18 and HIV immunogen induced significant CTL response and Ag-specific IFN-γ-secreting cells in mice (Staats et al., 2001).

Antibody levels and IFN-γ producing T cell responses were enhanced in mice immunized with genetic murine IL-18 and antigen intranasally (Lee et al., 2003). The survival rate of mice immunized with genetic IL-18 and antigenic DNA increased against vaginal challenge with a lethal dose of HSV-1. The intranasal administration of murine IL-18 with HA induced the production of Th1- and Th2-type cytokines, systemic IgG and mucosal IgA (Kayamuro et al., 2010b). Moreover, high levels of CTL were elicited in the presence of IL-18 and HA, protecting mice against a lethal influenza virus infection. Further analysis showed the mucosal adjuvanticity of IL-18 was dependent on the activity of mast cells. Regarding the close association between activation of Th1 cells and IL-18, mucosal administration of IL-18 may be specific for preventing intracellular infections.

## FLT3L

Fms-related tyrosine kinase 3 ligand (Flt3L) stimulates the proliferation and differentiation of various blood cell progenitors, including T cells, B cells, NK cells and DCs (Drexler and Quentmeier, 2004). OVA specific mucosal and plasma immune responses were significantly induced by intranasal administration of OVA and plasmid encoded murine Flt3L in comparison with OVA alone, leaning to a Th2 type (Kataoka et al., 2004). CD4^+^ T cells from the spleen and CLNs were highly proliferative and higher levels of IL-2 and IL-4 production were significantly accumulated in mice adjuvanted with Flt3L. The numbers of CD11c^+^ DCs in the mucosal effector sites, such as NPs, NALT and SMG, were remarkably enhanced due to nasal application of the expression plasmid encoding the Flt3L gene. Nasal administration of OVA and plasmid encoding mouse Flt3L enhanced OVA-specific mucosal and plasma antibodies and elicited Th2 immune response (Fukuiwa et al., 2008). Plasmacytoid DCs and CD8^+^ DCs were activated and expanded in the presence of plasmid encoding Flt3L. Mice nasally immunized with OVA and adenovirus expressing murine Flt3L exhibited high levels of OVA-specific mucosal and plasma antibodies as well as hyper-proliferation of OVA-specific CD4^+^ T cell and OVA-induced IFN-γ and IL-4 production in NALT, CLN and spleen (Sekine et al., 2008). OVA-specific CTL response was also enhanced in the spleen and CLN, and the number of CD11b^+^CD11c^+^ DCs expressing high levels of costimulatory molecules was preferentially increased in NALT, which migrated to mucosal effector lymphoid tissues. A single intratracheal application of human Flt3L dose dependently increased DC and T lymphocytes (CD4^+^ and CD8^+^) in rats, with a maximum on day 3 (Pabst et al., 2003). The cells in the lung interstitium and the bronchoalveolar space were affected by the administration of Flt3L, and subsequent intratracheal application of tetanus toxoid after the local Flt3L stimulation enhanced levels of antigen specific IgA and IgG in the lung.

Mice orally immunized with human Flt3L and CT were resistant to subsequent CT challenge in the model of ligated intestinal loops (Williamson et al., 1999). Flt3L-treated mice exhibited significantly elevated levels of CT-specific IgA Ab titers in both intestinal fluid and serum. The number of dendritic cells were increased in NALT with the nasal application of human Flt3L (Kodama et al., 2010). The co-administration of Flt3L and P6 protein of non-typeable *Haemophilus influenzae* induced P6-specific nasal wash IgA and serum IgG, antibody-producing cells and enhanced *Haemophilus influenzae* clearance from the nasopharynx in mice. Moreover, the longevity of vaccination was prolonged by nasal application of Flt3L and P6. Intranasal administration of pneumococcal surface protein A (PspA) and plasmid encoding the murine Flt3L increased levels of PspA-specific secretory IgA and IgG Ab responses that were correlated with elevated numbers of CD8^+^ and CD11b^+^ DCs and IL-2- and IL-4-producing CD4^+^ T cells in the NALT and CLNs (Kataoka et al., 2011). Correspondingly, numbers of CFU in the lungs, airway secretions and blood were markedly reduced when mice were nasally challenged with *Streptococcus pneumoniae* WU2. In conclusion, mucosal administration of Flt3L activates the interconnection between innate and adaptive immunity, which elicits both mucosal and systemic immune responses.

## CCR7 LIGANDS

C-C chemokine receptor type 7 (CCR7) and its ligands direct the migration of mature DCs and establish a functional microenvironment to prime naive T cells. A recent work demonstrated that intranasal administration of plasmid encoding murine Epstein-Barr virus-induced molecule 1 ligand chemokine (ELC) and HIV-1 gp140 significantly enhanced gp140-specific systemic and mucosal Ab responses in mice (Hu et al., 2013). Further analysis showed pELC co-delivery resulted in an increase of CCR7^+^ CD11c^+^ cells in mesenteric lymph nodes and both CCR7^+^ CD11c^+^ cells and CCR7^+^ CD3e^+^ cells in spleen.

Nasal and intragastric administration of genetic CCR7 ligands with herpes simplex virus 2 DNA plasmid stimulated distal mucosal IgA responses, and CD4^+^ T helper cell proliferation and CD8^+^ T cell-mediated CTL activity (Eo et al., 2001b). The number of dendritic cells was enhanced by co-delivery of CCR7 ligands in secondary lymphoid tissue. Secondary lymphoid tissue chemokine (SLC) specifically increased the production of Th1-type cytokines (IL-2 and IFN-γ) and ELC elicited the yield of both Th1-type and Th2-type (IL-4) cytokines (Eo et al., 2001b). Further analysis showed co-delivery of CCR7 ligands with antigen generated a high CTL population that was capable of rapid expansion following infection with HSV (Toka et al., 2003). The symptom was relieved and the survival rate was increased in CCR7 ligands vaccinated mice under the lethal dose challenge of HSV McKrae strain. In summary, the migration of DCs induced by CCR7 ligands enhances the antigen uptake at mucosal sites and antigen presentation at lymphatic tissues, thus efficaciously inducing immune responses.

## MIPS AND MCPS

Genetic application of CC chemokines macrophage inflammatory protein 1β (MIP-1β) and monocyte chemotactic protein 1 (MCP-1) primed Th2-type immune response, while the CXC chemokine MIP-2 and the CC chemokine MIP-1α elicited Th1-type pattern. Nasal approach of murine MIP-1α elicited significant increases in anti-OVA IgG1 and IgG2b Ab titers followed by IgG2a and IgG3 in mice (Lillard et al., 2003). Moreover, the production of IFN-γ, Th1 and CD8^+^ CTL responses were promoted in the presence of MIP-1α. Intranasal administration of murine MIP-1β induced antigen specific systemic IgG1 and IgG2b and mucosal IgA. Both Th1 and Th2 responses were distinctively induced by MIP-1β. Co-inoculation of murine MIP-1α expressing plasmid and HIV DNA vaccine increased not only systemic and mucosal antibodies, but also CTL activity and delayed type hypersensitivity in mice (Lu et al., 1999). The low ratio between IgG1 and IgG2a indicated the dominated Th1 response in the presence of MIP-1α.

The function of antigen-presenting cells and expressions of costimulatory molecules (B7-1 and B7-2) were up-regulated in the presence of murine MIP-1α, whereas Th1-type CD4^+^ T-cell-mediated adaptive immunity was enhanced by murine MIP-2 via increasing secretion of IFN-γ from activated NK cells (Eo et al., 2001a). In comparison with MIP-1β and MCP-1, mucosal genetic co-transfer of MIP-2 or MIP-1α with genetic antigen rendered recipients (mice) more resistant to HSV intravaginal infection. Nasal application of MIP-3α with P6 protein of nontypable *Haemophilus influenzae* (NTHi) induced an increase in the number of dendritic cells in murine NALT and P6-specific nasal wash IgA and serum IgG titers (Kodama et al., 2011). Accordingly, the clearance of NTHi was enhanced in MIP-3α immunized group.

## RANTES

RANTES (regulated on activation, normal T cell expressed and secreted) is an inflammatory chemokine that promotes the accumulation and activation of several types of leukocytes, including CD4^+^ T cells and macrophages (Baggiolini and Dahinden, 1994). Intravaginally co-administered murine RANTES-expressing plasmid (pRANTES) with antigen efficaciously expressed at the vaginal tissues, and induced more vaginal IgA and serum IgG immune responses than CT adjuvant in mice (Oh et al., 2003). Intranasal administration of plasmids carrying *env* and *rev* genes of HIV-1 and encoding murine RANTES induced significantly higher titers of serum HIV-1-specific IgG, IgG2a and fecal IgA antibodies than antigen DNA vaccination alone (Xin et al., 1999b). This vaccinated combination also increased HIV-1-specific CTL activity and delayed-type hypersensitivity in mice. Nasal co-administration of murine RANTES and the antigen initiated and enhanced Ag-specific humoral and cellular immune responses in both mucosal and systemic compartments (Lillard et al., 2001). Furthermore, nasal delivery of RANTES induced proliferative and Th1- (IL-2 and IFN-γ) type responses, and RANTES was proved to up-regulate CD28, CD40L and CD30 expression on both resting and activated T lymphocytes. The unique T cell activation by RANTES may facilitate vaccinations to achieve desirable T cell mediated immune responses.

## ANTIMICROBIAL MOLECULES

As chemotactic antimicrobial peptides for T cells, defensins exert adjuvant activity on the induction of antigen-specific immune response by nasal co-administration with antigens. Antigen-specific IgG1 followed by IgG2b and IgG2a was promoted by addition with human defensins, indicating the development of both Th-1 and selected Th-2 type immune responses. Furthermore, antigen specific CD4^+^ Th cell responses and cytokines of IL-5 and IL-6 were observed in the presence of defensins, corresponding to the Th1 and Th2 responses (Lillard et al., 1999a). Nasally co-administered human neutrophil peptide (HNP) defensins with protein antigens enhanced systemic immunity, however HNPs provided inhibitory signals for mucosal B and T cells. Mice intranasally immunized with HNPs displayed antigen specific proliferative responses of CD4^+^ and high production of IFN-γ, IL-5, IL-6 and IL-10, indicating HNPs elicited both Th1 and Th2 immune responses. In comparison with mice immunized with only OVA, human HNP-1, HBD1 and HBD2 induced significantly higher OVA-specific serum IgG, including IgG1 and IgG2b subtypes (Brogden et al., 2003). Moreover, the additive HNP-1, HNP-2 and HBD2 yielded lower IFN-γ and HBD1 enhanced the production of IL-10 in mice. Oral administration of cathelicidin LL-37 and conjugated antigen effectively evoked the antigen-specific systemic and mucosal immune responses, especially virus neutralization antibodies (Jang et al., 2013). Further analysis exhibited the number of germinal centers were significantly induced by chemotactic effect of LL-37 in both Peyer’s patch and mesenteric lymph node. In addition, strong Th1- and Th17-skewed immune responses were elicited through CD11c^+^ CD70^+^ cell activation in Peyer’s patch.

In comparison with BCG alone, delayed type hypersensitivity response was enhanced in mice orally administrated with lactoferrin and BCG (Chodaczek et al., 2006). Oral administration of lactoferrin increased the frequency of neutrophil precursors in the peripheral blood and decreased the spontaneous production of IL-6 and TNF-α by unstimulated blood cell cultures in human trials (Kruzel and Zimecki, 2002). Furthermore, patients subjected to thyroid surgery were treated with lactoferrin, exhibiting increased immune responsiveness. Beside the direct inhibition on pathogens, above antimicrobial molecules are also efficacious mucosal adjuvants, attracting further experimental and clinical trials on vaccine development.

## OTHER INNATE MUCOSAL ADJUVANTS

The above listed innate molecules have been well defined as mucosal adjuvants, however other endogenous molecules are reported to prime immune response with limited literatures. Lymphotactin (Lptn), belonging to C chemokine family, is predominantly produced by NK, CD8^+^ T cells and γδ TCR^+^ intraepithelial lymphocytes. Nasal administration of murine Lptn with protein antigens promoted both mucosal and systemic immunity (Lillard et al., 1999b). Mice immunized with antigen and Lptn displayed higher antigen-specific proliferative responses and increased synthesis of IFN-γ, IL-2, IL-4, IL-5, IL-6 and IL-10 of CD4^+^ T cells. This indicated that Lptn induced mixed Th1 and Th2-type responses. Eotaxin is a potent chemoattractant for eosinophils via the eotaxin receptor CCR-3. Intranasal administration of eotaxin caused chemotaxis of eosinophils with a clinically symptomatic inflammatory response in the human nasal mucosa, which was accompanied with the increase of nasal NO, contributing to oxidative stress (Hanazawa et al., 2000). Thymic stromal lymphopoietin acted as a potent mucosal adjuvant for HIV-1 gp140 vaccination in mice (Van Roey et al., 2012). The use of murine TSLP as an adjuvant skewed both the cellular and humoral immune responses towards Th2 cells in mice.

The intranasal administration of murine IL-33 with HA induced the production of Th1- and Th2-type cytokines, systemic IgG and mucosal IgA (Kayamuro et al., 2010b). Nasal approach of IL-33 and HA induced high levels of CTL, efficaciously protecting mice against a lethal influenza virus infection. Like IL-18, some activity of IL-33 is related to mast cells. B-cell activating factor (BAFF) secreted by stromal cells is crucial for survival and maturation of B cells (Batten et al., 2000; Kalled, 2006). Antigen specific systemic/mucosal antibodies were significantly enhanced in mice administrated with replication-deficient adenovirus vector expressing murine BAFF and heat-killed *P. aeruginosa* intranasally, reducing murine mortality under lethal dose challenge of *P. aeruginosa* (Tertilt et al., 2009). Moreover the spatial and temporal proximity with antigens were unnecessary for the application of BAFF as the immune booster.

## COMBINED INNATE ENDOGENOUS ADJUVANTS

Although many innate endogenous molecules are evaluated as mucosal vaccine adjuvants, combined adjuvants are required to induce desired immune responses. Co-administration of the DNA vaccine with murine IL-12- and GM-CSF-expressing plasmids induced high levels of HIV-specific antibodies (Okada et al., 1997). Moreover, CTLs and delayed type hypersensitivity were increased when both chemokines were administered to mice intranasally. Intranasal administration of adenovirus encoding murine IL-5 and IL-6 acted additively to enhance local mucosal IgA and IgG antibody responses (Braciak et al., 2000). Correspondingly viral specific antibody-secreting cells presented high frequency in lung lymphocytes derived from mice immunized with IL-5 and IL-6 expressing adenovirus.

Combinations of two or three cytokines including murine IL-1α, IL-12, IL-18 and GM-CSF plus HIV immunogen efficaciously enhanced mucosal IgA, induced CTL lytic activities, increased Ag-specific IFN-γ-secreting splenic cells and CD8^+^ in the peripheral blood (Staats et al., 2001). The antipeptide serum IgG and mucosal IgA titers were significantly induced by the combination of immunogens with multiple murine cytokines, e.g. IL-1α, IL-12 and IL-18 or IL-1α, IL-12 and GM-CSF (Bradney et al., 2002). Combination of human IL-1α and GM-CSF with antigen increased both mucosal and systemic antibodies by intranasal vaccinated route in macaques (Egan et al., 2004). The level of anti-DNP tear IgA was significantly enhanced in the lymphokine-treated rats co-administrated with antigen and murine IL-5 and IL-6 (Pockley and Montgomery, 1991). Rhesus macaques intranasally immunized with vaccinia virus Ankara carrying SIV DNA and plasmid expressing IL-2 and IL-15 or GM-CSF, IL-12 and TNF-α exhibited significant SIV-specific mucosal and systemic cell-mediated immunity (Manrique et al., 2011). Both vaccinated formulations significantly reduced the viral titer after intravaginal SIVmac251 challenge, corresponding to long survival time.

## CONCLUSIONS AND PERSPECTIVES

This review briefly describes the applications of innate endogenous molecules on developing mucosal adjuvants. The incorporation of these molecules significantly reduces the dose of antigens, enhances strong mucosal and systemic antibodies, elicits cell mediated immune response and eventually provides protections against pathogens or immune related diseases. The safety would be increased by the application of endogenous molecules instead of bacteria derived or synthetic materials. The mucosal route of vaccine delivery has been considered for a number of reasons including direct targeting of sites where the effects of given antigen or adjuvant are needed. Interestingly, some non-curable or recurrent infectious diseases could be effectively controlled by mucosal vaccinations as summarized in Table 1.

**Table 1:** Summary of innate endogenous adjuvants on mucosal immunization against HIV, HSV and influenza virus infections

Etiologies	Antigens	Adjuvants	Outcomes	References
HIV	Plasmid encoding *env* gp160 and *rev* genes	Plasmid encoding GM-CSF	Increase antibodies, enhance delayed type hypersensitivity	Okada et al. 1997
	gp120	TNF-α	Increase levels of antibodies	Kayamuro et al. 2010a
	C4-V3_IIIB_ and C4-V3_MN_	IL-1α	Increase antibodies and cellular immune responses	Staats et al. 2001
	Plasmid encoding HIV-1_IIIB_ *env* and *rev*	Plasmid encoding IL-2	Enhance Th1 dominant immune response	Xin et al. 1998
	Plasmid encoding *env* gp160 and *rev* genes	Plasmid encoding IL-4 or IL-12	Enhance Th1- and Th2-type responses and production of antibodies	Okada et al. 1997
	Plasmid encoding HIV-1_IIIB_ *env* and *rev*	Plasmid encoding IL-15	Increase the delayed type hypersensitivity response and CTL activity	Xin et al. 1999a
	C4-V3_IIIB_ and C4-V3_MN_	IL-18	Induce CTL response and Ag-specific IFN-γ-secreting cells	Staats et al. 2001
	Plasmid encoding gp140	Plasmid encoding ELC	Elicit systemic and mucosal Ab responses	Hu et al. 2013
	Plasmid encoding gp160 and *rev*	Plasmid encoding MIP-1α	Increase antibodies, CTL activity and delayed type hypersensitivity	Lu et al. 1999
	Plasmids expressing *env* and *rev* genes	Plasmid encoding RANTES	Increase antibodies, CTL activity and delayed-type hypersensitivity	Xin et al. 1999b
	gp140	Thymic stromal lymphopoietin	Facilitate cellular and humoral immune responses	Van Roey et al. 2012
	Plasmid encoding *env* gp160 and *rev* genes	Plasmid encoding IL12 + GM-CSF	Increase antibodies, CTL activity and delayed-type hypersensitivity	Okada et al. 1997
	C4-V3_IIIB_ and C4-V3_MN_	IL-1α, IL-12, IL-18 and GM-CSF	Enhance CTL response and Ag-specific IFN-γ-secreting cells	Staats et al. 2001
HSV	Attenuated HSV	Attenuated HSV expressing GM-CSF or IL-12	Protection against lethal dose viral challenge	Parker et al. 2006
	gD	IL-2	Enhance humoral and cellular immune responses, reduce the clinical scores and symptoms of Herpetic Keratitis	Niethammer et al. 2001; Inoue et al. 2002
	Plasmid encoding gB	Plasmid encoding IL-12	Induce humoral and cellular immune responses, protection against lethal dose challenge	Lee et al. 2003
	Plasmid encoding gB	Plasmid encoding IL-15	Enhance the Th1-dependent response and rapid elimination of virus	Toka et al. 2004; Toka and Rouse 2005
	gB	Plasmid encoding IL-18	Increase antibody levels, IFN-γ producing T cell responses and survival rate	Lee et al. 2003
	gB	CCR7 ligands	Increase CTL population and survival rate	Toka et al. 2003
	Plasmid encoding gB	Plasmid encoding MIP-2 or MIP-1α	Enhance cellular immune responses and protect from HSV challenge	Eo et al. 2001a
Influenza virus	Monovalent subunit influenza vaccine “Agrippal” IVR-116	IFN-α/β	Enhance humoral responses, prevent mice from live influenza virus infection and weight loss	Proietti et al. 2002; Bracci et al. 2005, 2006
	Inactivated monovalent A/Texas/91 (H1N1) vaccine	Type I IFN	Elicit high levels of antibodies, efficacious viral clearance	Couch et al. 2009
	Inactivated influenza vaccine	IFN-β	Increase survival rate	Cao et al. 1992
	HA derived from influenza virus A/New Caledonia/20/1999	IL-1α/β or IL-18 or IL-33	Enhance antibodies levels and survival rate	Kayamuro et al. 2010b
	Inactivated Influenza virus A/Ostrich/Denmark/72420/96	IL-2	Enhance mucosal immune responses	Zhang et al. 2009
	Influenza virus *ca* A/Sing/57-NS1_87	Virus expressing IL-2	Enhance mucosal and cellular immune responses, protect mice from lethal dose viral challenge	Ferko et al. 2006
	HA and N1A purified from influenza virus A/PR8/34	IL-12	Increase mucosal and systemic antibodies, protect mice from lethal dose viral challenge	Arulanandam et al. 1999

Cytokines are broadly used as mucosal adjuvants on the basis of their intrinsic characteristics, participating signal transduction of immune responses, activation of innate immunity and polarization of adaptive immunity. In use of the specific interactions between cytokines and corresponding receptors, desired immune responses are promptly and efficaciously primed. The relatively short half-life of recombinant homologues of cytokines has limited their use as vaccine adjuvants. These difficulties have been overcome by encapsulation into liposomes, virosomes and microparticles, and the use of cytokine expressing vectors co-administered with DNA vaccines. Moreover, the approach of DNA vaccines reduces the cost and increases the duration time of vaccinations. Beside cytokines, some other innate molecules are also identified as potent mucosal adjuvants recently.

Although some encouraging results on mucosal adjuvants have been achieved with different cytokines, important adverse effects are often associated with the large and repeated cytokine doses. Additionally, most cytokines are pleiotropic on the immune responses, sometimes eliciting unnecessary immune responses. The principle will be essential for rationally selecting vaccine adjuvants and application doses that induce potent immune responses in the absence of undesired effects. The homologies between human and non-human endogenous molecules vary differently, which affects the adjuvanticity in cross testing systems. Most innate mucosal adjuvants are evaluated on animal models, requiring further human trials for safety and efficacy of these potent molecules.

## ABBREVIATIONS

CCR7: CC chemokine receptor type 7
ELC: Epstein-Barr virus-induced molecule 1 ligand chemokine
Flt3L: Fms-related tyrosine kinase 3 ligand
gB: glycoprotein B
GM-CSF: granulocyte macrophage-colony stimulating factor
HA: hemagglutinin
HNP: human neutrophil peptide
IFNs: interferons
MCP-1: monocyte chemotactic protein 1
MIP-1β: macrophage inflammatory protein 1β
PspA: pneumococcal surface protein A
SLC: secondary lymphoid tissue chemokine
TNF: tumor necrosis factor
TT: tetanus toxin
